# Hyaluronic acid-functionalized bilosomes for targeted delivery of tripterine to inflamed area with enhancive therapy on arthritis

**DOI:** 10.1080/10717544.2019.1636423

**Published:** 2019-08-07

**Authors:** Hailing Yang, Zhenjie Liu, Yonglong Song, Changjiang Hu

**Affiliations:** a School of Pharmacy, Chengdu University of Traditional Chinese Medicine, Chengdu, China;; b School of Pharmacy, Guangxi University of Chinese Medicine, Nanning, China;; c Department of Pharmacy, Anhui Medical College, Hefei, China

**Keywords:** Tripterine, hyaluronic acid, bilosomes, targeted drug delivery, arthritis

## Abstract

Arthritis treatment has been challenging because of low drug exposure to the articular cavity. This study was intended to develop hyaluronic acid (HA)-functionalized bilosomes for targeted delivery of tripterine (Tri), an antiphlogistic phytomedicine, to the inflamed joint via ligand-receptor interaction. Tri-loaded bilosomes (Tri-BLs) with cationic lipid (DOTAP) were prepared by a thin film hydration method followed by HA coating to form HA@Tri-BLs. HA@Tri-BLs were then characterized by particle size (*PS*), entrapment efficiency (*EE*), and structural morphology. The *in vitro* drug release, hemocompatibility test and cellular uptake were performed to examine the formulation performances of HA@Tri-BLs. The *in vivo* pharmacokinetics and antiarthritic efficacy were evaluated in arthritic models, respectively. The obtained HA@Tri-BLs possessed a *PS* of 118.5 nm around with an *EE* of 99.56%. HA@Tri-BLs exhibited excellent cellular uptake and targeted delivery efficiency for Tri, which resulted in elongation of circulatory residence time and enhancement of intra-arthritic bioavailability (799.9% relative to Tri solution). The *in vivo* antiarthritic efficacy of HA@Tri-BLs was also significantly superior to uncoated Tri-BLs that gave rise to obvious inflammation resolution. Our findings suggest that HA-functionalized bilosomes are a promising vehicle for articular delivery of antiphlogistic drugs to potentiate their efficacy.

## 1. Introduction

Tripterine (Tri), also known as celastrol, is a natural compound derived from the root cortex of *Tripterygium wilfordii* (TW) with a diversity of biological activities, which is one of the effective ingredients against rheumatoid diseases in medicaments containing *TW.* Modern pharmacological studies show that Tri possesses strong antioxidative, antiangiogenic and antirheumatic effects (Ng et al., 2019). However, the insoluble nature of Tri in water greatly limits its bioaccessibility and clinical efficacy (Qi et al., 2014; Zhang et al., 2014). In order to enable formulation of Tri, a variety of nanocarriers have been developed for optimizing its oral or systemic delivery, including lipid-based nanoparticles (Chen et al., 2012; Li et al., 2015), polymeric nanoparticles (Sanna et al., 2015; Yin et al., 2017), albumin nanoparticles (Guo et al., 2017), liposomes (Soe et al., 2018), phytosomes (Freag et al., 2018), and micelles (Zhao et al., 2018). These delivery systems specific for Tri mostly focus on pharmacokinetic improvement and antitumor applications. To date, the unique merit of Tri in anti-arthritis has not been fully exploited due to lack of suitable delivery vehicles.

Bilosomes are flexible and deformable vesicles composed of phospholipid and bile salt that structurally resemble liposomes (Ahmad et al., 2017). They are (quasi) metastable in membrane organization due to insertion of bile salt molecules that lowers the phase transition temperature (Bnyan et al., 2018), making them ultra-flexible and highly deformable under the physiological temperature. Bilosomes belong to a type of transfersomes with the bile salt as the edge active molecule. In the case of transfersomes, the edge active molecules are normally surfactants that can alter the phase transition temperature of phospholipid bilayer (Hussain et al., 2017). Bilosomes have been broadly explored for bioavailability enhancement of various therapeutic agents (Shukla et al., 2016; Ahmad et al., 2017; Jain et al., 2017). Bilosomes also offer an alternative option for delivering active compounds to the poorly permeable tissues, such as the skin and the joint capsule. Although bilosomes possess many advantages as peroral and percutaneous delivery carriers, their suitability as a systemic delivery vehicle is compromised owing to lack of specificity and easy sequestration by the reticuloendothelial system (RES). Therefore, the surface engineering of bilosomes becomes particularly vital to potentiate and attenuate a candidate payload. Hyaluronic acid (HA), a major component of extracellular matrix, is the most important glycosaminoglycan in the synovial fluids, which plays a crucial role in the protection of articular cartilage (Litwiniuk et al., 2016). It has been proven that the regulating effects of HA on inflammation, cellular migration, and angiogenesis are completed via specific HA receptors (Misra et al., 2015), such as CD44 and RHAMM. CD44 is ubiquitously expressed on the surface of various pathologic cells with multiple functions in the inflammatory process (Pandey et al., 2017). It is highly interesting to take advantage of the homing nature of HA toward inflammation to construct inflammation-targeted drug delivery systems.

In this study, a bilosomal delivery system targeting to arthritis was developed based on the HA homing strategy used for systemic delivery of Tri to enhance its antiarthritic efficacy. We prepared HA-coated Tri-loaded bilosomes (HA@Tri-BLs) by thin film hydration method and characterized them with particle size, entrapment efficiency and morphology. The *in vitro* release, hemocompatibility, cellular uptake, *in vivo* pharmacokinetics and biodistribution, and *in vivo* antiarthritic effect of HA@Tri-BLs were evaluated and compared with uncoated Tri-loaded bilosomes (Tri-BLs) as well as free Tri.

## 2. Materials and methods

### 2.1. Materials

Tripterine was obtained from DESITE Biotechnology Co., Ltd (Nanjing, China). Soybean phosphatidylcholine (SPC, 95%) and 1, 2-stearoyl-3-trimethylammonium-propane (DOTAP) were provided by Avanti Polar Lipids, Inc. (Alabama, USA). Sodium deoxycholate (SDC), hyaluronic acid (HA), Hoechst 33258 and 3, 3’-dioctadecyloxacarbocyanine perchlorate (DiO) were purchased from Aladdin Reagents (Shanghai, China). Deionized water was prepared by a Milli-Q water purifier (Molsheim, France). Chlorpromazine, simvastatin, genistein, HUTCH-1 (monoclonal anti-CD44 antibody, rabbit), ethylisopropyl amiloride (EIPA) and Lantraculin A were purchased from Sigma-Aldrich (Shanghai, China). HPLC-grade methanol was supplied by Sinopharm Chemical Reagent Co., Ltd (Shanghai, China). All other chemicals were of analytical grade and used as received.

### 2.2. Preparation of tri-loaded bilosomes

Tri-loaded bilosomes (Tri-BLs) were prepared by the thin film hydration technique (Ahad et al., 2018). In brief, SPC, DOTAP and drug were dissolved in a round bottom flask with chloroform. In the case of HA-free bilosomes (as a reference formulation), DOTAP was not requisite for use. The organic phase was removed under reduced pressure using a rotatory evaporator, resulting in formation of a thin lipid film. After complete removal of residual organic solvent, the film was hydrated against an appropriate volume of phosphate buffer saline (PBS, pH 7.4) containing SDC. The resulting hydrated coarse bilosomes were homogenized with the microfluidizer (Nano DeBee, MA, USA) for 5 circles at 10,000 psi. To produce HA-coated Tri-BLs, Tri-BLs were incubated with HA in the bulk solution by which HA-functionalized Tri-BLs (HA@Tri-BLs) formed as a result of electrostatic interaction. The formulation factors affecting the formulation performances (*PS* and *EE*) were investigated, including the ratio of drug/lipids (DOTAP + SPC in a fixed ratio of 1/9) and the concentration of SDC in the aqueous phase upon hydration.

### 2.3. Characterization of tri-BLs

The particle sizes of Tri-loaded bilosomes (both Tri-BLs and HA@Tri-BLs) were measured using a Malvern laser particle analyzer (Nano ZS, Worcestershire, UK) at 25 °C. The bilosomes were duly diluted with deionized water and then placed in a disposable cuvette. After equilibrium for 120 s, the samples were subjected to laser diffraction for particle size analysis based on the dynamic light scattering principle. The same samples were proceeded for Doppler velocimetry test to determine their ζ potentials.

The morphology of Tri-BLs was observed with a Tecnai 10 transmission electron microscopy (TEM) (Philips, Eindhoven, Netherlands). The bilosomes, also diluted with deionized water, were dropped onto a carbon-coated copper grid and fixed by dehydration under a heating lamp. The fixed nanoparticles were then transferred to the TEM probe for inspection. Morphologic micrographs of Tri-BLs were taken at the acceleration voltage of 50 kV.

### 2.4. Determination of entrapment efficiency and drug loading

The entrapment efficiency (*EE*) of HA@Tri-BLs was measured by the centrifugal ultrafiltration with HPLC analysis (Deng et al., 2019). Freshly prepared HA@Tri-BLs were firstly centrifuged at 5000 rpm for 5 min to precipitate the possible unencapsulated species. Then, the upper part of nanosuspensions were carefully withdrawn and centrifuged against a centrifugal filter device (Amicon® Ultra-0.5, MWCO 50 kDa, Millipore) to collect the filtrate. Free Tri in the filtrate was quantified by HPLC established below. *EE* and drug loading (*DL*) were calculated according to Equations (1) and (2):

(1) $$\mathrm{EE}(\%)=(1-M_{\mathrm{fre}}/M_{\mathrm{tot}})\times 100\%$$

(2) $$\mathrm{DL}(\%)=(M_{\mathrm{tot}}-M_{\mathrm{fre}}/M_{\mathrm{tot}}+M_{\mathrm{exc}})\times 100\%$$

where *M*_fre_, *M*_tot_ and *M*_exc_ denote the amounts of free Tri, total Tri in the system and excipients used in the formulation, respectively.

Tri quantification was performed on an Agilent 1200 HPLC system (Santa Clara, CA, USA) using a Kromasil ODS C_18_ column (5 μm, 4.6 mm × 250 mm). A mobile phase consisting of 90% methanol and 10% water with 0.25% phosphoric acid (v/v) was utilized to elute the samples. The elution was monitored at 425 nm.

### *2.5. In vitro* release study

The release of Tri from Tri-BLs and HA@Tri-BLs was studied through a reverse dialysis technique (Zhang et al., 2015). In brief, aliquots of Tri-BLs or HA@Tri-BLs equal to 2.5 mg of Tri were put into 200 mL of pH 7.4 phosphate buffered saline (PBS) in a dissolution cup, in which sodium dodecyl sulfate (SDS) was added as a solubilizer (0.5%, w/v). The ready-to-use dialysis tubes (Spectra/Por^®^ Float-A-Lyzer^®^ G2, MWCO 100 kDa) loading blank medium were then placed in the dissolution cups for reverse dialysis. At predetermined intervals, 100 μL of medium was withdrawn from the dialysis tube. Tri concentration in the medium was analyzed by HPLC as described above, and the *in vitro* release curve was plotted based on the accumulative release percentage versus time.

### 2.6. Hemolysis and coagulation test

The hemolysis and coagulation of HA@Tri-BLs were tested according to the general rule 1148 of China Pharmacopeia 2015. Fresh blood was taken from the rat’s eyeball. A 2% suspension of erythrocytes was prepared using the rat blood after removal of fibrinogens by adsorption with glass pellets and dilution with 0.9% NaCl solution. Then, 0.3 mL of HA@Tri-BLs and 2.2 mL of 0.9% NaCl solution were added into 2.5 mL of 2% erythrocyte suspensions followed by incubation for 3 h at 37 °C. If the solution in the test tube is transparent and appears red, and no or just a small amount of erythrocytes precipitate at the bottom, it indicates the occurrence of hemolysis. If brownish red flocculates appear in the solution that cannot be re-dispersed after inversion for 3 times, it indicates that there is blood coagulation taking place.

### 2.7. Cellular uptake test

The cellular uptake of Tri-loaded bilosomes were investigated in RAW264.7 cells, a murine macrophage line. The cells were cultured in a RPMI-1640 medium containing 10% FBS (Gibco, CA, USA) at 37 °C in 5% CO_2_. When the cells grew to 80 ∼ 90% confluence, they were eligible for the cellular uptake test. Tri solution (dissolve in 75% ethanol), Tri-BLs and HA@Tri-BLs were diluted to 10 μg/mL with the culture medium and introduced into the cell wells for coincubation. To elucidate the uptake mechanism of HA@Tri-BLs, the uptake of HA@Tri-BLs was specially measured in the presence of various cytosis inhibitors or competitor, including hypertonic sucrose (400 mM), chlorpromazine (25 μM), simvastatin (5 μM), genistein (200 μM), free HA (25 μM), HUTCH-1 (10 μg/mL), EIPA (50 μM) and Lantraculin A (2.5 μM) (Deng et al., 2017). The intracellular drug concentration was determined using HPLC by lysing the cells with RIPA lysis buffer and corrected by the protein content with BCA protein assay kit.

To compare the cellular internalization capability of Tri-BLs and HA@Tri-BLs, fluorescent Tri-BLs and HA@Tri-BLs were prepared using 1-oleoyl-2-[6-[(7-nitro-2-1,3-benzoxadiazol-4-yl)amino]hexanoyl]-3-trimethylammonium propane (fluorescent DOTAP, Avanti) instead of part of SPC. Fluorescent Tri-BLs and HA@Tri-BLs were added in RAW264.7 cells and incubated for 0.5 h at 37 °C. The media were removed and the cells were washed with pH 7.4 PBS thrice. The cells were then immobilized by 4% paraformaldehyde and visualized under a confocal laser scanning microscopy (CLSM) (LSM700, Zeiss, Oberkochen, Germany) followed by nuclei staining with Hoechst 33258.

### 2.8. Pharmacokinetics and biodistribution studies in arthritic rats

The pharmacokinetics and tissue distribution of free Tri, Tri-BLs and HA@Tri-BLs were studied in arthritic rats. Sprague–Dawley (SD) rats weighing 250 ± 20 g were used to establish the arthritic model. The rats were acclimatized for 20 days in a standard animal room and provided with food and water *ad libitum*. On the 21st day, the rats were received injection of Freund’s complete adjuvant (FCA) containing 10 mg/mL of *Mycobacterium tuberculosis* (Sigma-Aldrich) at the right foot (Jia and He, 2016). The experimental rats were observed for days until the arthritis arose. If the right ankle shows acute inflammatory swelling within 24 h and obvious inflamed nodes appear in the fore and lateral limbs, it indicates success of model establishment. The established arthritic rats were intravenously injected with Tri solution (dissolved in 75% ethanol solution and diluted with deionized water), Tri-BLs and HA@Tri-BLs via the jugular vein at a dose of 10 mg/kg. After administration, aliquots of blood (∼250 μL) were continuously sampled from the caudal vein into heparinized tubes at predetermined intervals.

Another three groups of arthritic rats were applied for biodistribution study to analyze the *in vivo* drug distribution of Tri solution, Tri-BLs and HA@Tri-BLs. At 0.5, 1, 2 and 4 h after injection, mice were sacrificed, and the heart, liver, spleen, lung, and kidney as well as the joint were excised or harvested. The drug in the blood and tissue samples was extracted twice with ethyl acetate from the prepared plasma and tissue homogenates (Zhang et al., 2014). The extracted drug solution was then condensed and reconstituted in 50 μL of methanol for HPLC analysis with gossypol as an internal standard. The experimental protocols settled were reviewed and approved by the Animal Care and Use Committee of Chengdu University of Traditional Chinese Medicine.

### 2.9. Anti-arthritic effect of tri-BLs in arthritic mice

Male DBA/1 mice were used to construct collagen antibody-induced arthritis (CAIA) model. The mice were subcutaneously injected at the base of the tail with 100 μL of emulsions composed of 2 mL of bovine type II collagen (1 mg/mL) in 2 mL of FCA. On the 15th day of the first injection, a second intensive injection (50 μL of inducer) was carried out at the base of the tail. After 21 days of the first injection, the mice were evaluated for their arthritic index (AI) by visual inspection. The severity of arthritis was scored on a scale of 0 to 4 (0 = normal; 1 = minor erythema of toes; 2 = moderate erythema and swelling of paws; 3 = remarkable edema with limited motion of the joint; and 4 = severe edema with joint stiffening). Mice with an AI equal to 4 were selected for use in the *in vivo* experiment of anti-arthritic efficacy. The established arthritic mice were treated with saline, Tri-BLs and HA@Tri-BLs by caudal injection every three days for a total run of 5. The body weight of mice was recorded three days after each treatment, and the inflammation level was evaluated by calculating the average AI of extremities (Chen et al., 2017).

At the end of the treatment, the mice were sacrificed, blood was sampled from the eye socket, and the plasma was immediately prepared and stored at −18 °C until analysis. The inflammatory mediators reflecting the inflammatory level were measured using their ELISA kits (ExCell Bio, Shanghai, China) as per the manufacturers’ instructions, including tumor necrosis factor α (TNF-α), interleukin-6 (IL-6), and nitric oxide (NO). In addition, three mice were randomly selected from each treatment group and normal control group. Ankle joints of them were dissociated and fixed in 4% paraformaldehyde for two days. After decalcification, the ankle joints were prepared into paraffin sections and dyed with hematoxylin and eosin (H.E.) for histological check. The evolution of inflammation occurring in the joint was assessed by the histological responses.

## 3. Results and discussion

### 3.1. Preparation and characterization of tri-BLs

Bilosomes are novel lipid-based vesicles analogous to liposomes but incorporating a bile salt into the lipid bilayer instead of cholesterol. The preparation of bilosomes can refer to the preparative processes of liposomes (Ahad et al., 2018; D'Elia et al., 2019), such as thin film hydration method and melt method. The thin film hydration method represents a lightly exercisable technique under a lower temperature for production of bilosomes. However, the formulation variables have great effects on the formation of uniform vesicles. In this study, the factors affecting the *PS* and *EE* involved the ratio of drug/lipids and the SDC level in the aqueous phase. Figure S1 presents the effects of formulation variables on these two indices. The ratio of drug/lipids had great impact on *PS* and *EE* of Tri-BLs. High drug dosage in the formulation resulted in formation of larger nanoparticles, which may be attributable to elevated phase transition temperature due to interfusion of more Tri that impedes the curvature of lipid film. A drug/lipids of 1:10 was appropriate for the preparation of Tri-BLs before HA coating, which could produce nanovesicles smaller than 100 nm. Meanwhile, a low drug/lipids ratio was also favorable for drug entrapment that enabled Tri to be fully incorporated into the phospholipid bilayer. The bile salt level in the aqueous phase upon hydration showed elusive implications on *PS* and *EE* of Tri-BLs. At a moderate level of SDC, the nanovesicles prepared were the smallest and the *EE* was the highest. This may be connected with the surface activity and molecule aggregation of SDC. A low level of SDC has inferior capacity to reduce the surface tension for membrane curvature, while a high level of SDC tends to occur aggregation of molecules themselves.

**Figure 1.**
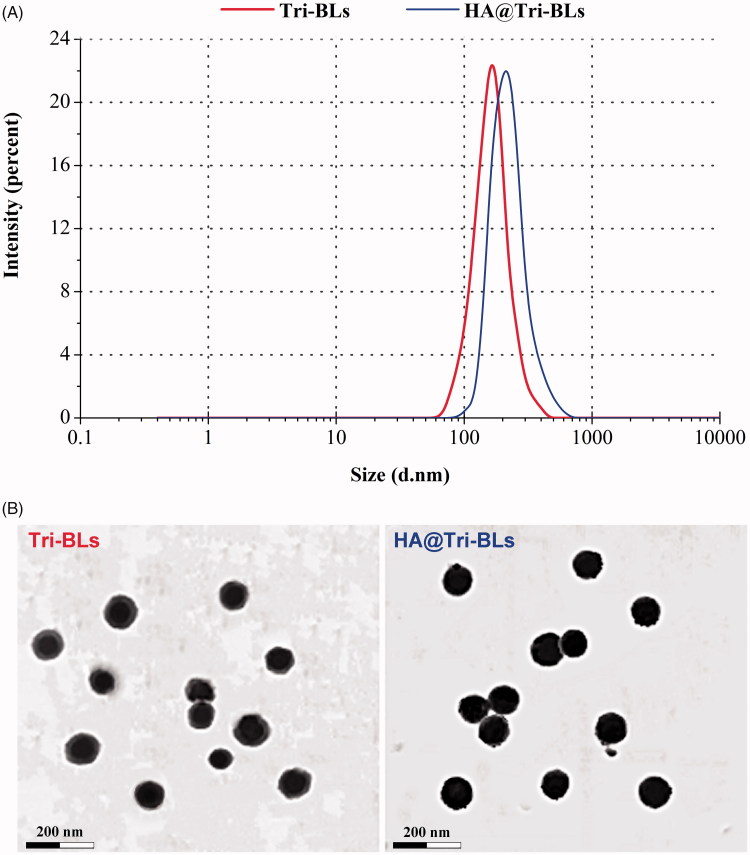
Particle size distribution (A) and TEM micrographs (B) of Tri-BLs and HA@Tri-BLs characterized by dynamic light scattering (DLS) and transmission electron microscope (TEM).

Considering the superiorities of small *PS* and high *EE* in drug delivery and cost control, the formulation was finally determined as 10 mg of Tri, 20 mg of DOTAP and 80 mg of SPC that were hydrated in 10 mL of SDC solution (2 mg/mL). Following preparation of Tri-BLs, Tri-BLs were functionalized with 10 mg of HA via electrostatic interaction between positively charged DOTAP and negatively charged HA. Tri-BLs and HA@Tri-BLs produced by the final formulation were 95.3 nm and 118.4 nm in *PS*, respectively. The *PS* of HA@Tri-BLs was larger than that of Tri-BLs, indicating occurrence of coating. Both Tri-BLs and HA@Tri-BLs displayed uniform *PS* distribution with a narrow polydispersity index (PDI) (Figure 1(A)). The ζ potentials were measured to be 4.8 mv and −34.2 mv for them, respectively. This also illustrates that HA@Tri-BLs have been successfully prepared and the resulting HA@Tri-BLs are stable as colloidal dispersions. The *EE* and *DL* of HA@Tri-BLs were up to 99.56% and 8.15%, which can be ascribed to the extremely strong lipophilicity of Tri. TEM revealed that Tri-BLs and HA@Tri-BLs were spherical in morphology (Figure 1(B)), but an apparent electron-dense corona was present around HA@Tri-BLs.

### 3.2. *In vitro* drug release

**Figure 2.**
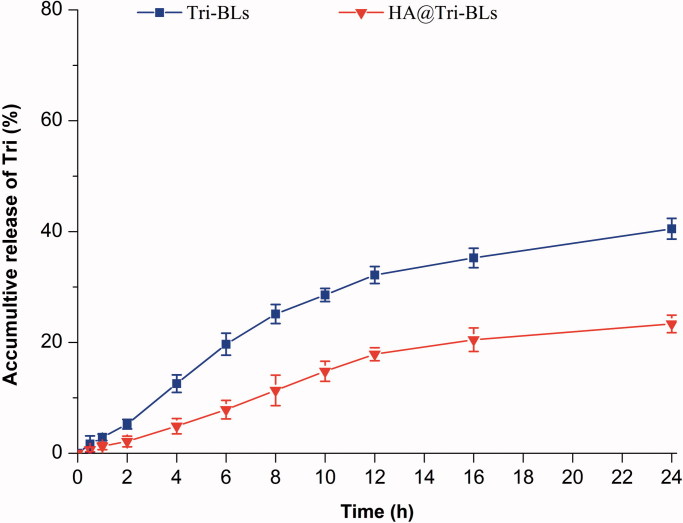
*In vitro* release profiles of Tri from Tri-BLs and HA@Tri-BLs in pH 7.4 phosphate buffered saline (PBS) based on the reverse dialysis method.

The *in vitro* release profiles of Tri from Tri-BLs and HA@Tri-BLs in pH 7.4 PBS are shown in Figure 2. Both Tri-BLs and HA@Tri-BLs exhibited a sustained release of Tri in the physiological pH buffer. The accumulative drug release nonlinearly increased with the release time, illustrating that the drug release follows the Fick’s law of diffusion where the attenuation of concentration gradient with the time slows down the release rate. For Tri-BLs, the accumulative release percentages were 25.13% at 8 h and 40.51% within 24 h, respectively. Owing to high hydrophobicity, Tri tends to be detained in the lipid bilayer of bilosomes and thus exhibits slower release in the medium. On the other hand, less drug release or no drug release is advantageous for realization of targeted drug delivery via nanovesicles, since off-target release will lead to side effects and/or adverse reactions. Of note, Tri release from HA@Tri-BLs was significantly slower than from Tri-BLs. This may be associated with HA coating that increases the thickness and viscosity of the diffusion layer. The accumulative release of Tri was merely 11.35% and 23.24% at the time of 8 h and 24 h for HA@Tri-BLs. It could be envisioned that the great majority of Tri would be incorporated in HA@Tri-BLs during circulation in the body after injection. This creates favorable conditions for site-specific release of the cargo with the navigation of the target ligand. The marginal drug release provided by HA@Tri-BLs enables Tri to be transported to the inflammatory area through integral vesicles whereby to potentiate the therapeutic action of this bioactive compound (Sun et al., 2019).

### 3.3. Hemocompatibility of HA@Tri-BLs

Hemocompatibility of intravenous formulations is a great concern for their safe application. After incubation with 2% erythrocyte suspensions for 3 h, HA@Tri-BLs did not result in cytolysis or coagulation of erythrocytes. The results show that there are no hemolysis and coagulation effects associated with HA@Tri-BLs. The excipient of HA used to coat Tri-BLs is a substance naturally present in the human body, thus HA-coated vesicles as injectable nanocarriers are normally highly biocompatible.

### 3.4. Uptake of tri-BLs in RAW264.7 cells

**Figure 3.**
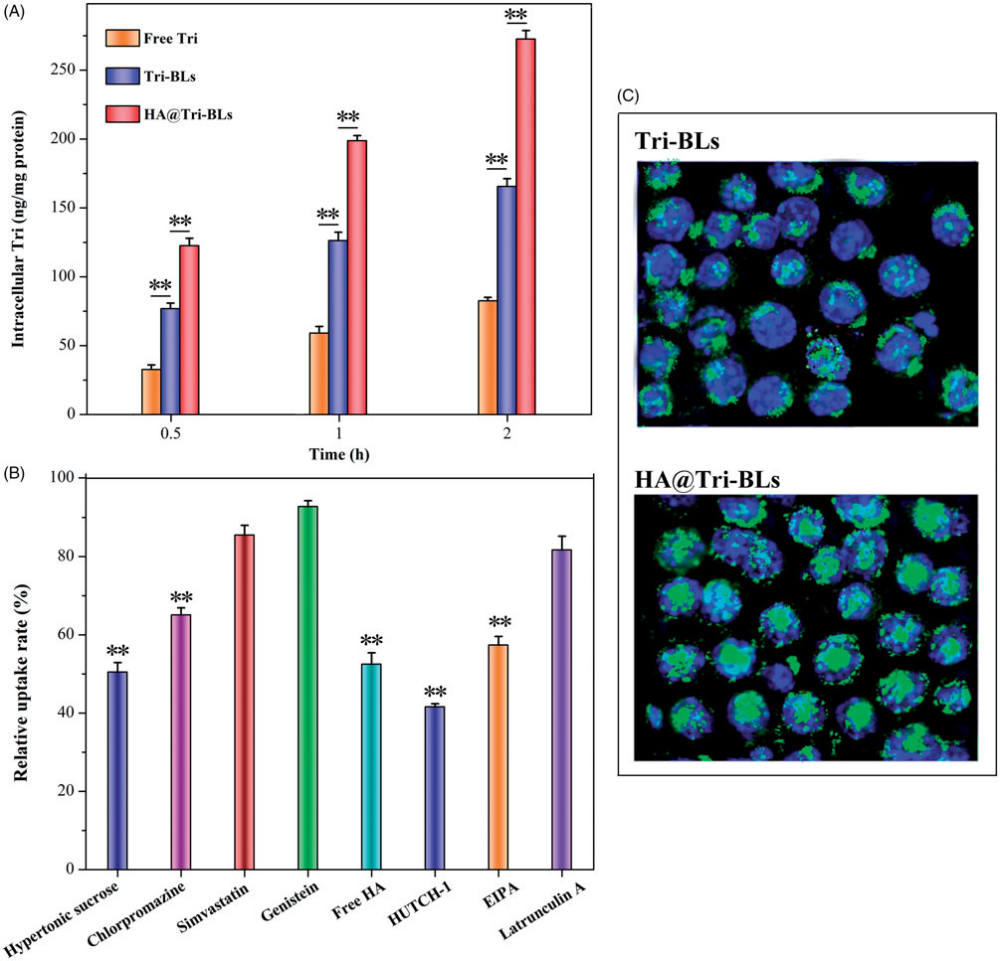
Cellular uptake, trafficking pathway and internalization investigation on RAW264.7 cells: (A) cellular uptake of free Tri, Tri-BLs and HA@Tri-BLs at different incubation time quantified by the intracellular Tri content (Statistically, paired t-test, **p < .01, significantly different each other); (B) relative uptake rate of HA@Tri-BLs in the presence of various cytosis inhibitors compared to the control (no inhibitor); and (C) cell internalization of Tri-BLs and HA@Tri-BLs characterized by CLSM (Statistically, paired *t*-test, **p < .01, significantly different from the control).

Figure 3(A) shows the cellular uptake of free Tri, Tri-BLs and HA@Tri-BLs in RAW264.7 cells. Three kinds of Tri modalities displayed a time-dependent cellular uptake, among which free Tri presented the minimal uptake level. The low cellular uptake of free Tri is perhaps related to its cytotoxicity that easily occurs cellular efflux. There is evidence indicating that Tri is a cytotoxic compound against a variety of cell lines (Yadav et al., 2018). When formulated into bilosomes, the cellular uptake of Tri was improved as revealed in the case of Tri-BLs. At three investigated time points, the uptake amounts increased by almost once. Encapsulation into bilosomes shields the undesired properties of Tri that facilitates it to internalize into the cells. By contrast, HA@Tri-BLs resulted in higher cellular uptake of Tri than Tri-BLs. We believe that it is related to HA functionalization of Tri-BLs, which escalates the cellular uptake by HA receptor-mediated influx. On this point, it can be judged from the relative uptake rates of HA@Tri-BLs in the presence of various cytosis inhibitors (Figure 3(B)). The addition of free HA and HUTCH-1 (a monoclonal anti-CD44 antibody) significantly inhibited the cellular uptake of HA@Tri-BLs, causing a reduction of 47.5% and 58.4% in uptake, respectively. In addition, hypertonic sucrose, chlorpromazine and EIPA also inhibited the cellular uptake of HA@Tri-BLs to different extents. These chemical agents are normally used as biological inhibitors of nonspecific, specific clathrin-mediated endocytosis and macropinocytosis. Therefore, it can conclude that the cellular trafficking of HA@Tri-BLs involved in RAW264.7 cells is bound up with the pathways of clathrin and CD44 receptor-mediated endocytosis along with macropinocytosis in mechanism.

The intracellular transport capability of HA@Tri-BLs can be appreciated from the difference in the cellular internalization between Tri-BLs and HA@Tri-BLs (Figure 3(C)). Intense fluorescence staining took place in RAW264.7 cells upon incubation with HA@Tri-BLs compared to Tri-BLs. There were considerable vesicles-associated fluorescence distributing within the cytoplasm, even into the nucleus as indicated by the colocalization. The cellular internalization of Tri-BLs was markedly inferior to HA@Tri-BLs where there were just a small quantity of cells stained by fluorescent Tri-BLs. High affinity and permeability to the inflammation-related cells allow HA@Tri-BLs to be specifically delivered to the inflammatory area, thereby achieving a targeted therapy (Palvai et al., 2017).

### 3.5. Bioavailability and biodistribution of tri-BLs in arthritic rats

**Figure 4.**
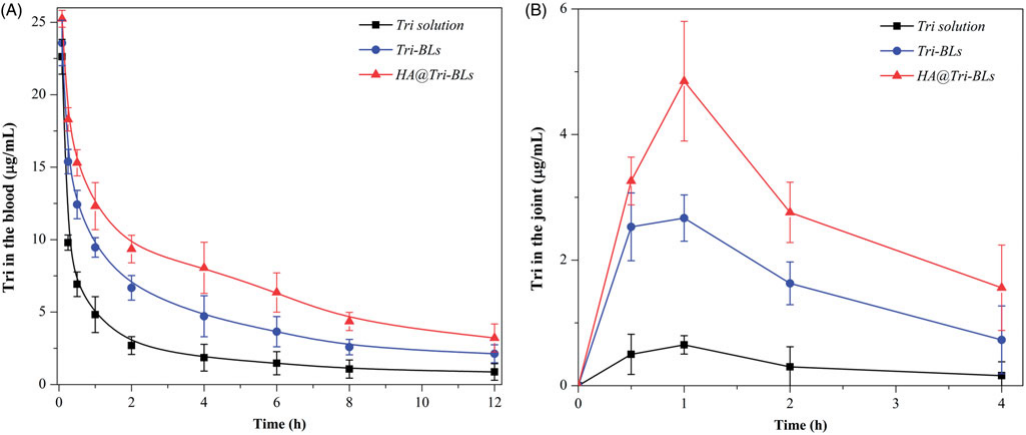
Pharmacokinetic profiles of Tri after injection of Tri solution, Tri-BLs and HA@Tri-BLs in arthritic rats (*n* = 6): (A) plasma drug concentration-time curves; and (B) intra-articular drug concentration-time curves.

The pharmacokinetic profiles of Tri in arthritic rats after injection of various Tri formulations are given in Figure 4 with main pharmacokinetic parameters summarized in Table 1. The formulation of Tri solution underwent prompt decline of drug concentration (Figure 4(A)). When encapsulated in bilosomes, the elimination rate of Tri was retarded up to a point, especially in the case of HA@Tri-BLs. This can be explained by complete exposure of Tri to metabolic enzymes and rapid excretion via the kidney *in vivo*. Encapsulation allows the drug to be released slowly and thus reduces the total body clearance. In comparison with Tri-BLs, HA@Tri-BLs enables Tri to be retained in the circulatory system for a longer time. This explicitly has something to do with HA coating on the outer layer of bilosomes that reduces complement opsonization and subsequent sequestration by RES to some extent (Alphandery, 2019). The maximum plasma drug concentration (*C*_max_), area under the plasma drug concentration-time curve from zero to infinity (*AUC*_0–∞_), apparent distribution volume (*V*), and total body clearance (*C*_L_) of HA@Tri-BLs were significantly different from Tri solution and Tri-BLs. Smaller *V* and lower *C*_L_ were provided with HA@Tri-BLs due to reduced distribution toward peripheral tissues and prolonged residence in the circulatory system. The targetability of HA@Tri-BLs could be estimated from the intra-articular drug concentration-time curve (Figure 4(B)). HA@Tri-BLs resulted in higher levels of Tri in the joint fluid compared with free Tri and Tri-BLs. The *C*_max_ and *AUC*_0–∞_ pertinent to the joints reached 4.854 μg/mL and 11.783 μg·h/mL, whereas they were merely 0.652 μg/mL and 1.473 μg·h/mL for Tri solution and 2.671 μg/mL and 7.075 μg·h/mL for Tri-BLs, respectively. The relative articular bioavailability of HA@Tri-BLs, calculated based on a non-compartment model, was up to 799.9% compared with Tri solution, significantly higher than that of Tri-BLs (480.3%) (Table 2). The difference in articular bioavailability between Tri-BLs and HA@Tri-BLs manifests that HA functionalization is fairly helpful to increase transport of Tri-BLs toward the joint. We assume that it is accomplished through CD44 receptors that are highly expressed on activated macrophages, which plays a crucial role in the pathophysiology of rheumatoid arthritis (Yu et al., 2019).

**Table 1.** Main pharmacokinetic parameters of Tri in arthritic rats after administration (*i*.*v*.) of Tri solution, Tri-BLs and HA@Tri-BLs.

Formulation	Tri solution	Tri-BLs	HA@Tri-BLs
*C*_max_ (μg/mL)	22.62 ± 0.71	23.57 ± 0.49	25.24 ± 0.57*
*AUC*_0−∞_ (μg/mL*h)	35.86 ± 0.53	76.19 ± 1.13	112.19 ± 0.85**
*T*_1/2_(h)	6.15 ± 0.22	5.98 ± 0.47	5.75 ± 0.38
*V* (L)	1.97 ± 0.35	1.03 ± 0.23	0.72 ± 0.16**
*C*_L_ (L/h)	0.279 ± 0.086	0.131 ± 0.054	0.089 ± 0.063**
*MRT* (h)	7.073 ± 0.767	7.922 ± 0.681	8.075 ± 0.319

*C*_max_: maximum plasma concentration; *AUC*_0−∞_: area under the plasma concentration-time curve from zero to infinity; *T*_1/2_: half-life; *V*: apparent volume of distribution; *C*_L_: total drug clearance from plasma; *MRT*: mean residence time

Paired-*t* test, *p < .05, **p < .01, significantly different from Tri solution and Tri-BLs. Data expressed as mean ± SD (*n* = 6).

**Table 2.** Comparative pharmacokinetic indices of Tri in the joint after *i*.*v*. injection of Tri solution, Tri-BLs and HA@Tri-BLs.

Formulation	Tri solution	Tri-BLs	HA@Tri-BLs
*C*_max_ (μg/mL)	0.652 ± 0.146	2.671 ± 0.367	4.854 ± 0.674**
*AUC*_0−t_ (μg/mL*h)	1.473 ± 0.402	7.075 ± 0.213	11.783 ± 0.388**
*RBA*	/	480.3%	799.9%

*RBA*: relative bioavailability; *AUC*_0−t_: area under the plasma concentration-time curve from zero to the time of the last quantifiable concentration.

Paired-*t* test, **p < .01, significantly different from Tri solution and Tri-BLs. Data expressed as mean ± SD (*n* = 6).

**Figure 5.**
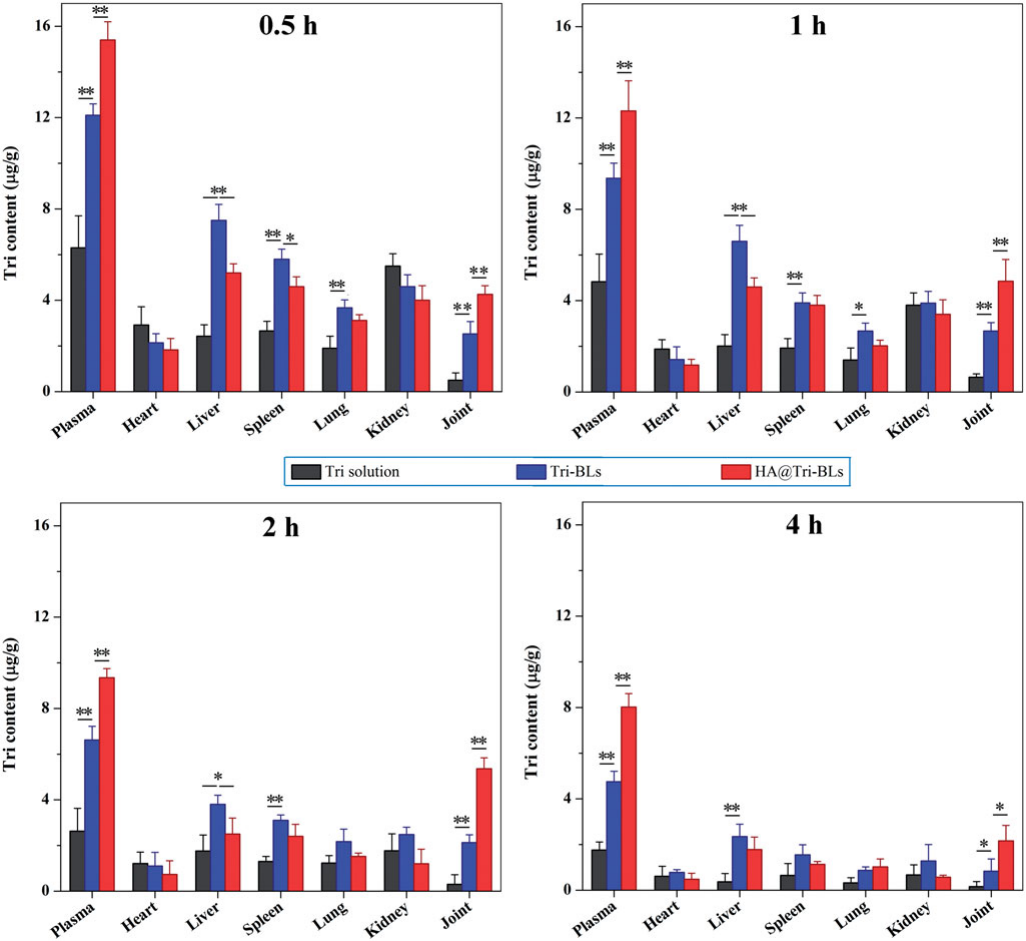
Biodistribution of Tri in the plasma, heart, liver, spleen, lung, kidney and joint after injection of Tri solution, Tri-BLs and HA@Tri-BLs for 0.5, 1, 2 and 4 h. Statistical analysis: paired *t*-test, **p < .01, HA@Tri-BLs significantly different from Tri solution and Tri-BLs.

The biodistribution of Tri in different formulation modalities with the time is depicted in Figure 5. As shown in the histograms, free Tri exhibited high distribution in the organs of heart and kidney besides the plasma; however, bilosomal Tri had high distribution in the liver, spleen and lung featured by RES, which may be related to the capture of vesicles by macrophages in the RES. Of note, Tri contents in the joints in groups of Tri-BLs and HA@Tri-BLs were markedly higher than that of free Tri. It can be attributable to the prolonged circulatory half-life of bilosomal Tri and nonspecific endocytosis of vesicles by inflammatory cells that result in high accumulation into the arthritic joints. In addition, the intra-articular Tri level in the group of HA@Tri-BLs was significantly higher than Tri-BLs at all investigated time points. These results suggest that HA can effectively facilitate Tri-BLs transport into the arthritic joints. Coating Tri-BLs with HA brings about much greater accumulation of Tri in the inflamed joints than HA-free Tri-BLs. The underlying reasons lie in the extravasation effect of vesicles via the leaky vasculature into the inflammatory joint and specific endocytosis mediated by CD44 receptors (Chen et al., 2015).

### 3.6. Therapeutic efficacy of tri-BLs in the arthritis model

**Figure 6.**
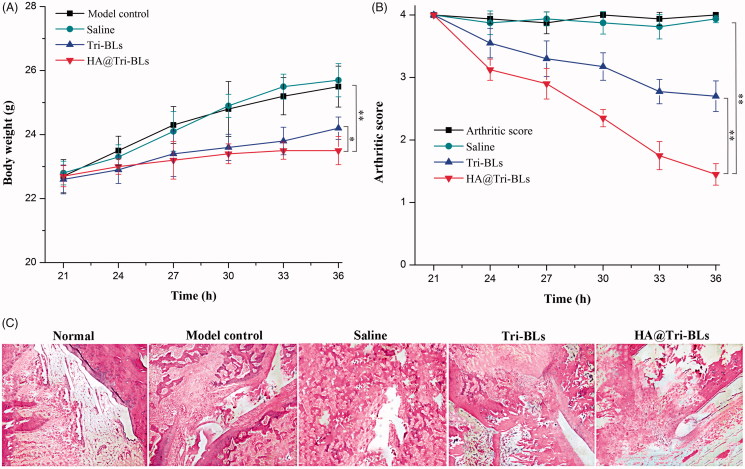
Ant-arthritic efficacy of HA@Tri-BLs *in vivo*: (A) Body weight change in different treatment groups; (B) Average arthritic scores of mice assessed by the hind paw following treatment with saline, Tri-BLs and HA@Tri-BLs (data shown as mean ± SD, *n* = 6); (C) Histopathological changes in the joints stained with H.E. of arthritic mice (magnification 200×). Statistical analysis: ANOVA, *p < .05, **p < .01, HA@Tri-BLs significantly different from Tri-BLs, saline and model group.

In this study, a CAIA model was utilized to evaluate the *in vivo* antiarthritic effect of HA@Tri-BLs (Fernandez-Zafra et al., 2019). The body weight and arthritic score of CAIA mice were traced following treatment, and the articular histomorphology was checked after treatment. HA@Tri-BLs maintained well the body weight of CAIA mice at a befitting level, significantly reduced the arthritic score, and effectively enhanced the therapeutic efficacy of Tri on inflammation compared with Tri-BLs (Figure 6). Due to extremely poor water-solubility, we failed to prepare the formulation of Tri solution for repeated administration to investigate the antiarthritic effect of free Tri. Like the model control, the mice treated saline all increased their body weight, perhaps due to on-going tissue edema as a result of aggravated inflammation. To some extent, Tri-BLs suppressed the body weight gain of mice, though HA@Tri-BLs exhibited more prominent bioactivity to balance the body weight of arthritic mice (Figure 6(A)). Likewise, HA@Tri-BLs resulted in more marked inflammation recession as reflected by the arthritic score of mice after treatment (Figure 6(B)). The daily activity of mice in the saline treatment group was still restricted, and the erythema and swelling around the joints did not relieve compared to the control group. Tri-BLs treatment alleviated the inflammatory lesion, getting the ankle joint and paw well to a moderate level of swelling. In comparison with Tri-BLs, HA@Tri-BLs gave rise to the optimal therapeutic efficacy that basically recovered the joint movement of mice from a limited state. The ankle swelling disappeared or significantly became light. These results indicate that HA@Tri-BLs are provided with superior targeted therapy for arthritis. We argue that this is associated with the high deformability of bilosomes and the specific affinity of HA@Tri-BLs to CD44 receptors largely expressed on inflamed tissues (Kosovrasti et al., 2016).

The subsided inflammation by HA@Tri-BLs was supported by the histomorphological change in the joint sections (Figure 6(C)). The model group exhibited apparent inflammatory characteristics, such as synovium hyperplasia, infiltration and pannus formation. Treatment with saline did not substantially alter these pathological features. Differently, Tri-BLs produced a certain antiarthritic effect that attenuated cell infiltration and pannus formation. In comparison with Tri-BLs, HA@Tri-BLs demonstrated a more potent anti-inflammatory activity, which markedly reduced the thickness of synovial cell lining and the level of bone damage. HA@Tri-BLs exhibited great potential to block the progression of inflammation and provide protection to the joint. This can be attributed to enhanced accumulation of Tri in the inflamed area with the assistance of CD44 receptor-specific nanovesicles.

### 3.7. Effects of tri-BLs on inflammatory mediators

TNF-α, IL-6 and NO able to reflect the inflammation level were selected as indicative mediators to assess the effect of HA@Tri-BLs on arthritis (Siu et al., 2018). These mediators play important roles in the pathological progression of various inflammatory diseases. The serumal levels of pro-inflammatory mediators in the CAIA mice following treatment with saline, Tri-BLs and HA@Tri-BLs are shown in Figure S2. The normal mice exhibited lower TNF-α, IL-6 and NO levels, while elevated levels of mediators appeared in the model mice, indicating successful establishment of arthritis model. After treatment, Tri-BLs and HA@Tri-BLs substantially reduced the serumal levels of TNF-α, IL-6 and NO of CAIA mice compared with the model group. However, treatment with saline failed to result in appreciable decline of inflammatory mediators. These results turn out that bilosomes enable the exertion of the drug’s bioactivity. By contrast, the inhibitory effect of HA@Tri-BLs on inflammatory mediators was stronger than that of Tri-BLs. HA@Tri-BLs exhibited the maximal capacity of downregulation toward TNF-α, IL-6 and NO. This can be explained by the enhanced exposure of Tri in the inflammatory site due to the CD44 receptor targeting of HA@Tri-BLs that inhibits the release of inflammatory cytokines.

TNF-α is a cell signaling cytokine involved in the process of inflammation that mediates the acute phase reaction. IL-6 can work as both a pro-inflammatory cytokine and anti-inflammatory cytokine. But, if overexpressed, it stimulates the immune response to aggravate the development of inflammation. NO also actively takes part in the pathogenesis of inflammation, which produces the anti-inflammatory effect under a normal physiology, whereas it induces inflammation upon overproduction under the pathological state. It is clinically significant for modulating abnormal production of TNF-α, IL-6 and NO whereby to relieve the inflammatory reaction and control the inflammatory development. Our designed HA@Tri-BLs showed superb *in vivo* regulating effect on various inflammatory mediators, which is potential for treating various inflammatory diseases, including but not limited to rheumatoid arthritis.

## 4. Conclusions

In this study, HA-functionalized bilosomes were engineered for targeted delivery of Tri to inflamed joint with the aim of enhancing the antiarthritic efficacy. The performance of HA@Tri-BLs as nanomedicine for arthritis treatment was examined. HA@Tri-BLs were readily prepared by coating bilosomes with HA via electrostatic complexation. Small particle size, high entrapment rate and sustained release of Tri were provided with HA@Tri-BLs. The *in vivo* pharmacokinetic studies revealed that improved circulatory residence time and enhanced systemic and intra-arthritic bioavailability of Tri were achieved through HA@Tri-BLs. As expected, HA@Tri-BLs resulted in excellent *in vivo* anti-inflammatory effect, significantly superior to HA-free Tri-BLs. It is shown that the enhanced antiarthritic efficacy in terms of HA@Tri-BLs can be attributable to high accumulation of Tri in the articular cavity due to HA-mediated transport. This work contributes an inspiration to targeted arthritis therapy taking advantage of HA ligand and flexible vesicles for specific delivery of anti-inflammatory agents.

## Supplementary Material

Supplemental Material
