# Supplementary material for: Hyaluronic acid-functionalized bilosomes for targeted delivery of tripterine to inflamed area with enhancive therapy on arthritis
Source: Drug Deliv. 2019 Aug 7;26(1):820–30. doi: 10.1080/10717544.2019.1636423 (PMC6713218; doi:10.1080/10717544.2019.1636423)
Supplement: Supplemental Material [file IDRD_A_1636423_SM5310.pdf]

Supporting information

Hyaluronic acid-functionalized bilosomes for targeted delivery of tripterine to inflamed area with enhancive therapy on arthritis

Hailing Yang [a,b], Zhenjie Liu [b], Yonglong Song [c], Changjiang Hu [a,*]

[a] School of Pharmacy, Chengdu University of Traditional Chinese Medicine, Chengdu 610072, China.

[b] School of Pharmacy, Guangxi University of Chinese Medicine, Nanning 530001 China.

[c] Department of Pharmacy, Anhui Medical College, Hefei 230601,China.

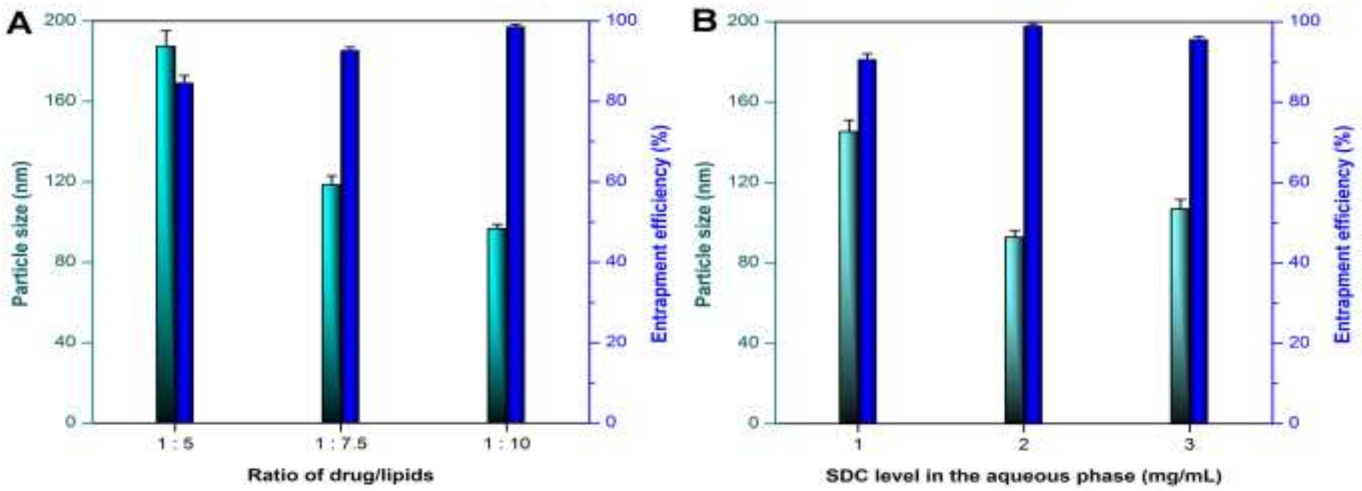

Figure S1. Formulation factors affecting the particle size and entrapment efficiency of Tri-loaded bilosomes: (A) the ratio of drug/lipids and (B) the level of sodium deoxycholate (SDC) in aqueous phase upon hydration.

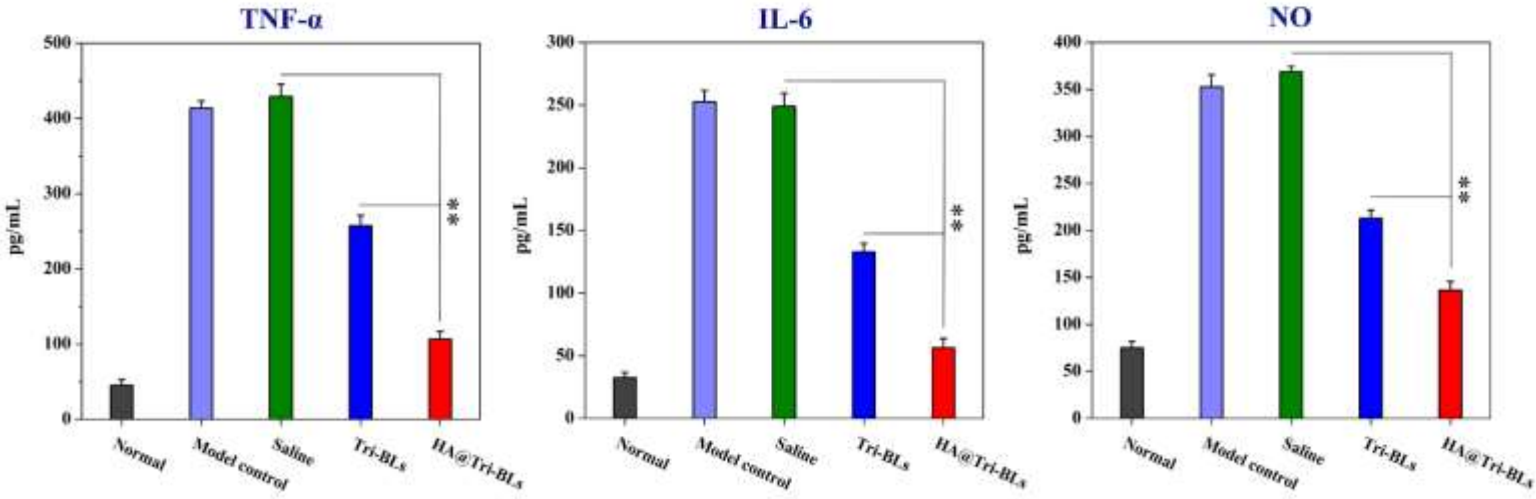

Figure S2. Serumal levels of inflammatory mediators (TNF-α, IL-6 and NO) in adjuvant-induced arthritic mice following treatment with saline, Tri-BLs and HA@Tri-BLs. Data expressed as mean ± SD (n = 3). Statistic analysis: Paired t-test, $^{**}P < 0.01$, HA@Tri-BLs significantly different Tri-BLs and saline.
